# P-1829. Low Hepatocellular Carcinoma Surveillance Among Cirrhotic Patients in a Mobile and Telehealth HCV Elimination Program

**DOI:** 10.1093/ofid/ofaf695.1998

**Published:** 2026-01-11

**Authors:** Jihad Slim, Kevin Leyden, Corey Rosmarin-DeStefano, Emily Levaggi, Sheena Duprey, Juan Torres, Anthony Raymond

**Affiliations:** Saint Michael’s Medical Center, Newark, NJ, USA, Newark, NJ; NJCRI, Newark, NewJersey; North Jersey Community Research Initiative, Newark, NewJersey; NJCRI, Newark, NewJersey; NJCRI, Newark, NewJersey; NJCRI, Newark, NewJersey; NORTH JERSEY COMMUNITY RESEARCH INITIATIVE, Newark, New Jersey

## Abstract

**Background:**

We evaluated the proportion of patients diagnosed with cirrhosis through our hepatitis C (HCV) elimination program in New Jersey who engaged in biannual hepatocellular carcinoma (HCC) screening. The program operates through a mobile van that collects initial bloodwork at drug addiction treatment facilities across New Jersey, followed by a telehealth consultation with an infectious disease physician for liver fibrosis staging and treatment recommendations.

**Methods:**

We conducted a cross-sectional observational study of patients diagnosed with liver cirrhosis who achieved sustained virologic response (SVR) through our HCV elimination program. Following SVR, intensive follow-up was provided by a registered nurse, case manager, 340B coordinator, and pharmacist. At the SVR visit, patients with cirrhosis were counseled to establish six-month HCC surveillance with a primary care provider or specialist. Patients diagnosed with cirrhosis on or before September 30, 2024, were contacted in April 2025 to assess their engagement in an HCC screening program.

**Results:**

Between June 1, 2021, and September 30, 2024, 1,897 patients with HCV were evaluated; 120 (6.3%) were diagnosed with cirrhosis. The median age was 55 years (34–72), and 91 (76%) were male. Among the 120 patients contacted, 67 (56%) were lost to follow-up, 3 (2.5%) had died, 3 (2.5%) were incarcerated, and 1 (0.8%) was hospitalized. Of the 46 reachable patients, 16 (35%) reported no follow-up, 20 (43%) reported PCP follow-up, and 8 (17%) reported subspecialist follow-up. None had received a six-month surveillance liver ultrasound.

**Conclusion:**

Despite targeted education, referrals, and case management, engagement in HCC surveillance among cirrhotic patients treated through a mobile and telehealth model remained low. Future efforts should prioritize developing intervention strategies to strengthen linkage to HCC surveillance after HCV cure.

**Disclosures:**

Jihad Slim, MD, FACP, gilead: Honoraria|merck: Honoraria|Thera: Honoraria|ViiV: Honoraria Kevin Leyden, RN, RN, Abbvie Inc.: Advisor/Consultant|Gilead Sciences: Advisor/Consultant|Gilead Sciences: Grant/Research Support

